# Chromosome-level genome assembly of the silver pomfret *Pampus argenteus*

**DOI:** 10.1038/s41597-024-03070-0

**Published:** 2024-02-23

**Authors:** Jiehong Wei, Yongshuang Xiao, Jing Liu, Angel Herrera-Ulloa, Kar-Hoe Loh, Kuidong Xu

**Affiliations:** 1 Institute of Oceanology, Chinese Academy of Sciences, Qingdao, China; 2 University of Chinese Academy of Sciences, Beijing, China; 3 Escuela de Ciencias Biológicas, Universidad Nacional, San José, Costa Rica; 4 Institute of Ocean and Earth Sciences, Universiti Malaya, Kuala Lumpur, Malaysia

**Keywords:** Genomics, Genome

## Abstract

*Pampus argenteus* (Euphrasen, 1788) is one of the major fishery species in coastal China. *Pampus argenteus* has a highly specialized morphology, and its declining fishery resources have encouraged massive research efforts on its aquacultural biology. In this study, we reported the first high-quality chromosome-level genome of *P. argenteus* obtained by integrating Illumina, PacBio HiFi, and Hi-C sequencing techniques. The final size of the genome was 518.06 Mb, with contig and scaffold N50 values of 20.47 and 22.86 Mb, respectively. The sequences were anchored and oriented onto 24 pseudochromosomes based on Hi-C data corresponding to the 24-chromatid karyotype of *P. argenteus*. A colinear relationship was observed between the *P. argenteus* genome and that of a closely related species (*Scomber japonicus*). A total of 24,696 protein-coding genes were identified from the genome, 98.9% of which were complete BUSCOs. This report represents the first case of high-quality chromosome-level genome assembly for *P. argenteus* and can provide valuable information for future evolutionary, conservation, and aquacultural research.

## Background & Summary

*Pampus argenteus* (Euphrasen, 1788; Fishbase ID: 491), also known as the silver pomfret, is a commercially important fish in the Northwest Pacific area that is widely distributed throughout the South China Sea to coastal Japan, Korea, and Russia^1,2^. It belongs to the family Stromateidae of the suborder Stromateoidei^3^, which was identified in Scombriformes according to a recent phylogenetic study^4^. This species is one of the major fishery species in coastal China, with harvests exceeding three million tons in 2016^5^. Overfishing and environmental changes have resulted in a noticeable decline in *P. argenteus* fishery resources in recent years^6,7^. The aquaculture of *P. argenteus* has made substantial progress, which in some ways compensates for the decline in fishery resources^8,9^. However, the industry is still facing many restrictions and issues owing to the high sensitivity of *P. argenteus* to injury and pathogenic infection during aquaculture and transportation^10^. Due to the medusivorous habit of *P. argenteus*^11^, its aquaculture greatly relies on fish bait composed of jellyfish and minced fish meat. Using fish bait leads to higher costs in water quality control and risking outbreaks of harmful pathogens, which have become one of the major bottlenecks in *P. argenteus* aquaculture, necessitating substitution with better formulated feeds^12^. However, the digestive and immune systems of *P. argenteus* are considered specialized for the digestion of jellyfish and tolerance of medusocongestin^13,14^. The inclusion of jellyfish in an artificial diet can significantly improve the growth performance and survival rate of *P. argenteus* larvae and juveniles^15^. The impact of changing fish bait to formulated feed on *P. argenteus* at different growth stages still requires further clarification. Clarifying the genetic basis of the physiological process of *P. argenteus*, particularly those related to the immune response^16^, intestinal enzyme activities^14^, stress responses^17^, etc., is becoming increasingly important for the future prospects of the aquaculture industry. However, the genome of *P. argenteus*, which represents the foundation of physiological responses^18^, has not yet been completely sequenced.

In addition to its fishery importance, *P. argenteus* is considered one of the most advanced species within Stromateoidei^19^. The dorsal and anal fin spines of *P. argenteus* are reduced into small blades, with a pelvic fin absent from its abdominal region. Stromateoidei is distinct from other Actinopterygii by having a unique pharyngeal sac immediately behind the last gill arch, which functions to fragmentize food^19^. The saccular structure of *P. argenteus*, which primarily feeds on small crustaceans and medusae, is smaller, more elongated, and densely covered within elongated tooth-like papillae; additionally, this species ably adapts to shredding rubbery tissue of jellyfish^19^. The pharyngeal sac is believed to be a key innovation for stromateiods, while the specialized shape of pharyngeal sac in the genus *Pampus* might bring further advantages and lead to its broad success in the Indo-Pacific region^19^. Clarifying the genetic basis for the formation of the pharyngeal sac is crucial to understanding the evolution of the genus *Pampus*.

**Fig. 1:**
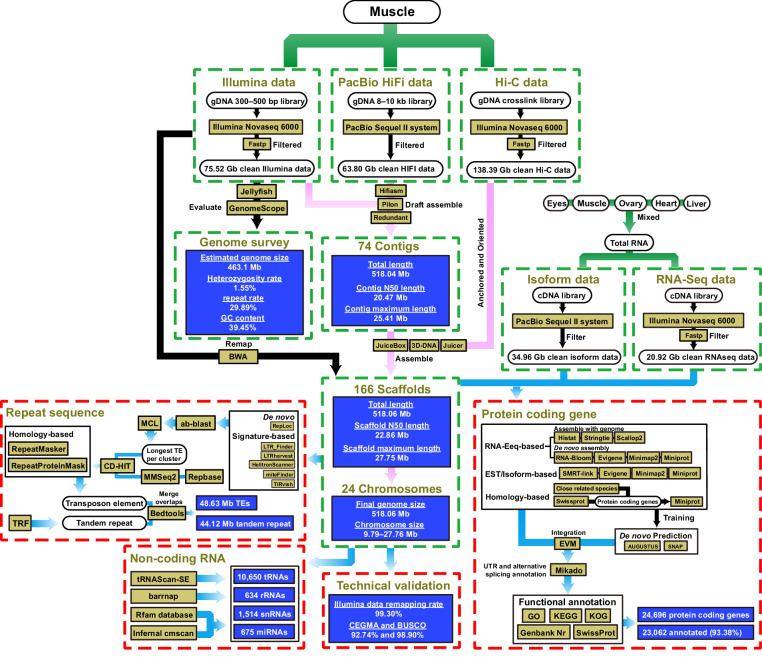
Workflow overview for the P. argenteus chromosome-level genome assembly.

**Fig. 2:**
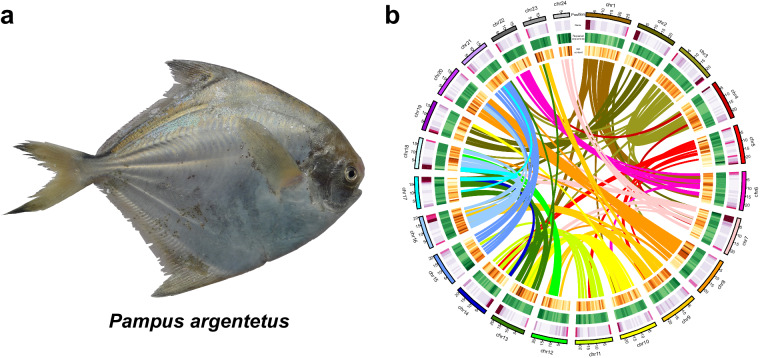
(**a**) A photo of *P. argenteus*; (**b**) Circos plot indicating gene density, repetitive sequences, GC content, and colinear relationship among chromosomes of the *P. argenteus* genome assembly.

In this study, a high-quality chromosome-level genome assembly of *P. argenteus* was generated by integrating multiple sequencing technologies, including Illumina sequencing, PacBio circular consensus sequencing (CCS), and Hi-C techniques (Fig. 1). The final assembly size for the *P. argenteus* genome was 518.06 Mb, with 97.30% of the contigs anchored to 24 chromosomes (Table 1 & Fig. 2). The contig and scaffold N50 lengths for the genome were 20.47 and 22.86 Mb, respectively. The genome consisted of 13.45% repeated sequences and 17.18% nod-coding genes. A total of 24,696 protein-coding genes were predicted, 93.38% of which were functionally annotated. Compared to the *Pampus* genome reported by AlMomin *et al*.^20^, the genome of *P. argenteus* generated herein was assembled into fewer and longer contigs and scaffolds (Table 1). More genes and repetitive regions were identified from this genome, with an average protein-coding gene length 7.5-fold greater than that of the previous version^20^. These results suggested that the genome developed in this study has a much higher integrity and quality. The chromosome-level genome assembly of *P. argenteus* will provide valuable information for establishing effective molecular markers for future conservation and aquaculture goals. The genome also represents the first case of high-quality chromosome-level genome assembly for stromateoids; this information could be an important reference for whole-genome sequencing of its close relatives, and, foreseeably, could become one of the foundations for exploring the genomic evolution and phylogeny of the Stromateoidei.

**Table 1:** Comparison of the *Pampus* genome assemblies in this study and the study of AlMomin *et al*.^20^.

	This study	AlMomin *et al*.^20^
Technologies	Illumina + PacBio CCS	Illumina
Clean data (Gb)	75.52 (Illumina)	76.43 (Illumina)
	63.80 (PacBio)	
Coverage	145.77 (Illumina)	58 × (Illumina)
	123.15 × (PacBio)	
Hi-C clean data (Gb)	138.39	—
Genome size (Mb)	518.06	350.05
Repeat rate (%)	13.57	11.06
GC content (%)	40.15	38.82
# Chromosomes	24	—
Chromosome size (Mb)	9.80–27.75	—
# Contigs	74	2,097,109
Total length (Mb)	518.04	694.94
Contig N50 (Mb)	20.47	0.0005
Contig Max (Mb)	25.41	0.0186
Average Length (Mb)	7.00	0.0003
# Scaffolds	166	298,141
Total length (Mb)	518.06	350.06
Scaffold N50 (Mb)	22.86	0.0016
Scaffold Max (Mb)	27.75	0.0331
Average Length (Mb)	3.12	0.0012
# Protein coding genes	24,696	16,322
Average gene length (bp)	14137.19	1,890.19
# non-coding genes	13,473	1,085
tRNAs	10,650	203
rRNAs	634	187
miRNAs	1,514	443
snRNAs	675	252

## Methods

### Sample collection

In 2021, a female *P. argenteus* specimen was caught from the wild for whole-genome sequencing using a fishing boat in Shengsi, Zhejiang Province, China. The specimen was identified based on the morphological descriptions of *P. argenteus* in Liu *et al*.^3^, who designated the *P. argenteus* neotype. Eye, muscle, ovary, heart and liver samples for DNA and RNA sequencing were isolated from the specimens immediately after they were caught. The samples were washed three times with phosphate-buffered saline (PBS), frozen in liquid nitrogen for three hours, and subsequently stored at −80 °C until DNA extraction. All the experiments were conducted under the approval and regulations of the Institutional Animal Care and Use Committee of the Institute of Oceanology, Chinese Academy of Science.

### Library construction, sequencing and data preparation

The Illumina, PacBio HiFi, and Hi-C data were obtained and used for generating a chromosome-level genome assembly of *P. argenteus*. For Illumina sequencing, total genomic DNA was isolated from muscle samples using the cetyltrimethylammonium bromide (CTAB) method^21^. The quality of the extracted DNA was assessed using a Qubit 2.0 (Thermo Fisher Scientific, USA) and a NanoDrop^®^ Series (Thermo Fisher Scientific, USA). For Illumina sequencing, a short-fragment library with an insert size of 300–500 base pairs (bp) was prepared using the NEBNext^®^ΜLtra™ DNA Library Prep Kit (New England Biolabs, USA) following the manual instructions. The library was purified with AMPure XP Beads (Beckman Coulter, USA) and then subjected to sequencing on an Illumina NovaSeq 6000 platform (Illumina, USA) to generate 150-bp paired-end (PE150) reads. After filtering in Fastp (v0.20.0)^22^, a total of 75.52 Gb of clean Illumina PE150 data were obtained, with Q20 and Q30 being 97.2% and 92.5%, respectively (Table 2). For PacBio CCS, total genomic DNA total genomic was extracted from muscle samples using the sodium dodecyl sulfate (SDS) method^23^. The high-molecular-weight gDNA was sheared to 8–10 kb using g-TUBEs (Covaris, USA). The HiFi library was then prepared using the SMRTbell prep kit 3.0 and sequenced in CCS mode on the PacBio Sequel II system (Pacific Biosciences, USA) following the manufacturer’s protocols. After the removal of low-quality reads and adaptors from the raw data, 63.80 Gb of clean HiFi data was retained, with Q20 and Q30 values of 99.9% and 54.78%, respectively (Table 2). Hi-C library preparation was performed with muscle tissue using a Frasergen Hi-C Kit (Frasergen, China) following the protocol instructions, which included crosslinking, lysis, fragmentation, repairing, biotin labeling, ligation, extraction, purification, and library construction. All the purification steps were performed using AMPure XP beads (Beckman Coulter, USA), while the biotin-labeled DNA was enriched with Pierce™ Streptavidin Magnetic Beads (Thermo Fisher Scientific, USA). The library was assessed with an Agilent 2100 Bioanalyzer (Agilent, USA) to determine a sufficient concentration and an insert size of 300–800 bp. The Hi-C library was subjected to sequencing on an Illumina HiSeq X Ten platform (Illumina, USA) to generate PE150 reads. After filtering in Fastp^22^, a total of 138.39 Gb of clean Hi-C data were obtained, for which the Q20 and Q30 were 96.57% and 90.54%, respectively (Table 2).

**Table 2:** Sequencing data used for the *P. argenteus* genome assembly.

	Illumina	PacBio	Hi-C
Clean data (Gb)	75.52	63.80	138.39
Sequencing depth (×)	145.77	123.15	267.13
GC content (%)	41.24	40.26	40.45
Q20 (%)	97.06	99.99	96.57
Q30 (%)	92.50	98.34	90.54

To assist in gene prediction, muscle, eye, ovary, heart, and liver tissues were pooled to obtain the transcriptome of *P. argenteus*. Total RNA was extracted from the pooled sample using a TRIzol reagent kit (Invitrogen, USA) following the manufacturer’s instructions. The quality and concentration of the extracted RNA were assessed using a NanoDrop^®^ Series (Thermo Scientific, USA) and an Agilent 2100 Bioanalyzer. For RNA-seq data, three cDNA libraries (i.e., Pa-op1, Pa-op2, and Pa-op3; Table 3) were prepared via total RNA extraction using the NEBNext® Ultra™ RNA Library Prep Kit (New England Biolabs, USA) and subsequently subjected to sequencing on an Illumina NovaSeq 6000 platform (Illumina, USA). After filtering via Fastp^22^, a total of 20.91 Gb of clean RNA-seq data were obtained from the five tissue samples (Table 3). For isoform data, a single cDNA library was reverse transcribed from the total RNA using the Clontech SMARTer PCR cDNA Synthesis Kit (Takara Bio, USA) following the manufacturer’s instructions. The PCR products were purified using AMPure XP Beads (Beckman Coulter, USA) and used for SMRTbell library construction via the SMRTbell Prep Kit 3.0. The library was sequenced and processed with the PacBio Sequel II system (Pacific Biosciences, USA). After filtering, a total of 34.96 Gb of isoform data were obtained (Table 3). The reference genome and protein-coding gene data of closely related species of *P. argenteus* [i.e., *Dunckerocampus dactyliophorus* (Bleeker, 1853)^24^, *Hippocampus zosterae* Jordan & Gilbert, 1882^25^, *Scomber japonicus* Houttuyn, 1782^26^, *Thunnus albacares* (Bonnaterre, 1788)^27^, and *T. maccoyii* (Castelnau, 1872)^28^] were downloaded from GenBank and subsequently used for gene prediction and comparisons.

**Table 3:** The transcriptomic data of *P. argenteus* used for gene prediction.

Type	RNA-seq data			Isoform data
	Pa-op1	Pa-op2	Pa-op3	
Raw data (Gb)	6.79	6.97	7.34	89.34
Clean data (Gb)	6.72	6.92	7.27	34.96
GC content (%)	47.14	47.39	47.03	48.21
Q20 (%)	98.22	98.23	98.16	93.43
Q30 (%)	94.64	94.67	94.5	88.44

The raw isoform data refers to the subread data generated in PacBio CCS, while its clean data is the CCS reads generated from the subreads.

### Genome survey

**Fig. 3:**
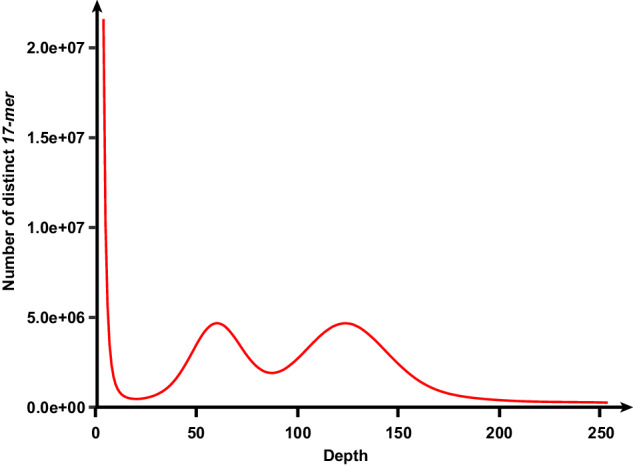
17-mer frequency distribution in the *P. argenteus* genome, the numbers of *k-mers* of each sequencing depth are indicated.

A survey of the *P. argenteus* genome was performed using the *k-mer* method. *K-mer* analysis was conducted using jellyfish (v2.2.6)^29^ with 75.52 Gb of Illumina data and the best K value of 17. After the removal of abnormal *k-mers*, 60,502,700,002 *k-mers* were yielded with a *k-mer* peak at a depth of 126.64 (Table 4 & Fig. 3). The genome size, heterozygosity rate, repetition rate, and GC content estimated from GenomeScope (v1.0.0)^30^ were 463.10 Mb, 1.55%, 29.89% and 39.45%, respectively (Table 4).

**Table 4:** Genome survey results.

K_*mer*_	Depth	N_*k-mer*_	Genome size (Mb)	Heterozygous rate (%)	Repeat rate (%)	GC content (%)
17	126.64	60,502,700,002	463.10	1.55	29.89	39.45%

### Chromosome-level genome assembly

**Fig. 4:**
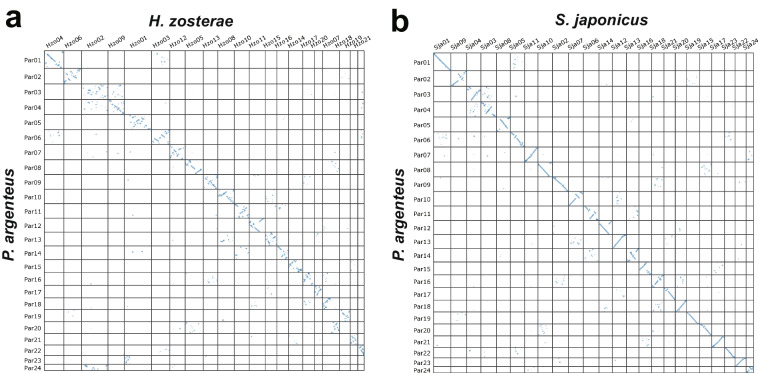
Dot plots showing the collinearities of *P*. *argenteus* with the syngnathiform (*H. zosterae*) and scombriform (*S. japonicus*) species.

The genome of *P. argenteus* was first assembled into 416 contigs with HiFi long-read data using the default parameters in Hifiasm (v0.16.1)^31^. The Illumina PE150 data were used to correct the contig assemblies in Pilon (v1.23)^32^. Finally, 74 non-redundant contigs with a total length of 518.04 Mb were obtained in Redundans (v14a)^33^. The contig N50 and maximum length were 20.47 and 25.41 Mb, respectively (Table 1). The clean Hi-C data were aligned to the genome assembly using BWA (v0.7.12)^34^. Reading depth and coverage were calculated in Picard (v1.129)^35^ and BEDtools (v2.25.0)^36^. To obtain the chromosome-level genome, clean Hi-C data were assembled with 74 contigs and adjusted using Juicer (v1.6)^37^, 3D-DNA (v180114)^38^ and JuiceBox^39^. Finally, the assembled sequences were anchored and oriented to 24 pseudochromosomes, ranging in size from 9.80–27.76 Mb (Table 1), which is congruent with the 24-chromatid karyotype reported by Liu *et al*.^40^. The total length of the chromosome-level assembly was 518.06 Mb, with a scaffold N50 of 22.86 Mb (Table 1 & Fig. 1). Therefore, the clean Illumina, PacBio HiFi, and Hi-C data had 145.77-, 123.15-, and 267.13-fold coverage of the *P. argenteus* genome, respectively (Table 2). A collinearity dot plot generated using MCScanX^41^ and SynVisio^42^ indicated clear genomic collinearity between *P. argenteus* and the scombriform species *S. japonicus*^26^ but scattered collinearity with the syngnathiform *H. zosterae*^27^, supporting the closer affinity of *P. argenteus* to the scombriform species (Fig. 4).

### Repeated sequence annotations

Tandem repeats were predicted using Tandem Repeats Finder (v4.10.0) (TRF)^43^. Transposable elements (TEs) were identified by a combination of signature-based, *de novo*, and homology-based approaches. *De novo* prediction was performed using RepLoc (v2021-3-12)^44^, and TEs shorter than 30 bp were removed from the results. Long terminal repeat retrotransposons (LTR-RTs) were identified using both LTR_FINDER (v1.0.2)^45^ and LTRharvest (v1.6.2)^46^. Miniature inverted-repeat transposable element (MITE) sequences were found by MiteFinder (v1.0.006)^47^. Helitron sequences were scanned by a HelitronScanner (v1.0)^48^. TIRvish (v1.6.2)^49^ was used to find terminal inverted repeat (TIR) sequences. All the predicted repetitive sequences were combined with the known repetitive sequence data in the Repbase database^50^ to generate the non-redundant repeat sequence library for *P. argenteus* using AB-BLAST (v3.0)^51^, MCL (v14–137)^52^, MMseqs. 2 (v13.45111)^53^ and CD-HIT^54^. The final repeat sequences in the *P. argenteus* genome were identified and classified by homology searching against the library using RepeatMasker (v4.1.2-p1)^55^ and RepeatProteinMask (v4.1.2-p1)^56^. In brief, 13.57% of the *P. argenteus* genome was annotated as repetitive elements, with 9.39% 9.39% TEs (48.64 Mb) and 8.51% tandem repeats (44.10 Mb) (Table 5).

**Table 5:** Repeat sequences of the *P. argenteus* genome annotated using different methods.

Methods	RepeatMasker	RepeatProteinMask	TRF	Combined
**(I) Transposon elements**	**40.30(7.78%)**	**16.18(3.12%)**	—	**48.64(9.39%)**
DNA	17.99(3.47%)	3.07(0.59%)	—	19.19(3.71%)
LTR	13.39(2.58%)	3.85(0.78%)	—	14.95(2.89%)
LINE	8.86(1.71%)	9.25(1.79%)	—	14.44(2.78%)
SINE	0.06(0.01%)	—	—	0.06(0.01%)
**(II) Tandem repeats**	**17.05(3.30%)**	—	**27.06(5.22%)**	**44.10(8.51%)**
Simple repeat	15.00(2.90%)	—	—	15.00(2.90%)
Low complexity	2.05(0.40%)	—	—	2.05(0.40%)
Tandem repeat	—	—	27.06(5.22%)	27.06(5.22%)
**(III)Unknown**	**2.16(0.42%)**	—		**2.16(0.42%)**
**Total**	**57.70(11.14%)**	**16.18(3.12%)**	**27.06(5.22%)**	**69.68(13.45%)**

Total length (Mb) and percentage (within bracket) of the *P. argenteus* genome for each type of repeat sequence are shown.

### Non-coding RNA annotation

For non-coding RNA annotation, tRNA and rRNA were predicted by tRNAScan-SE (v2.0.9)^57^ and barrnap (v0.9)^58^, respectively, while snRNA and miRNA were identified by aligning to the Rfam database^59^ with Infernal cmscan (v1.1.4)^60^. A total of 10,650 tRNAs, 634 rRNAs, 1,514 snRNAs, and 675 miRNAs were identified, comprising 17.18% of the *P. argenteus* genome (Table 6).

**Table 6:** Information of different types of non-coding RNA genes identified in the *P. argenteus* genome.

Type		Number	Total length(bp)	Percentage of genome
miRNA		675	59,026	1.14%
tRNA		10,650	389,423	7.52%
rRNA	Total	634	222,239	4.29%
	18 S	28	48095	0.93%
	28 S	26	115566	2.23%
	5.8 S	27	4061	0.08%
	5 S	553	58495	1.13%
snRNA	Total	1,514	219,261	4.23%
	CD-box	169	16187	0.31%
	HACA-box	73	10891	0.21%
	splicing	1259	189571	3.66%
	scaRNA	12	2555	0.05%
	Unknown	1	57	0.00%
Total		13,473	889,949	17.18%

### Protein-coding gene prediction and annotation

**Fig. 5:**
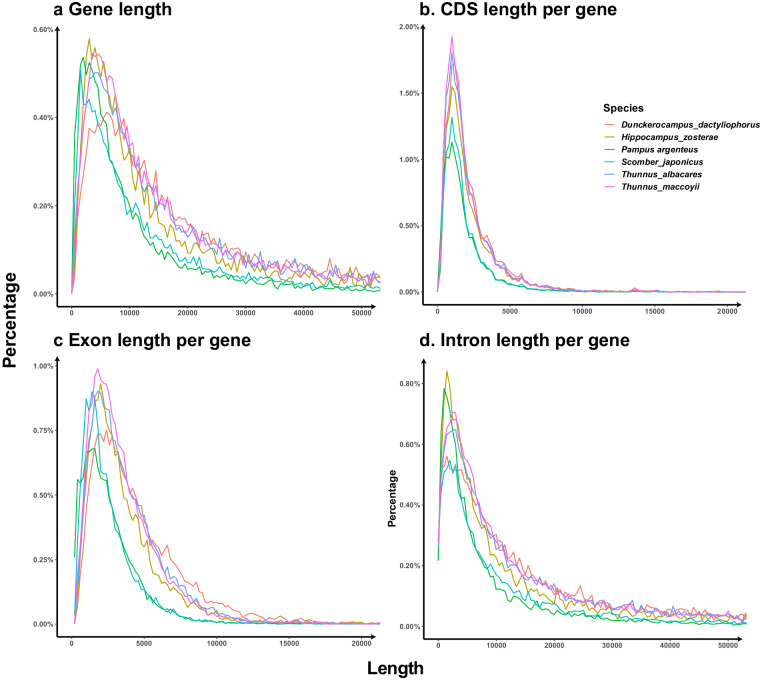
Comparisons of gene, CDS, exon, and intron lengths of *P. argenteus* and the five closely related species (*D. dactyliophorus*, *H. zosterae*, *S. japonicus*, *T. albacares* and *T. maccoyii*).

**Fig. 6:**
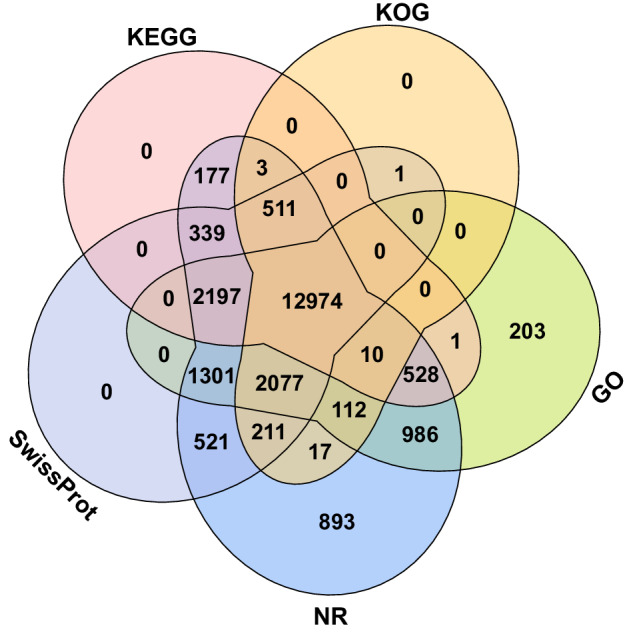
Venn diagram indicating number of genes annotated by different gene databases.

The protein-coding genes were predicted based on four different strategies, namely, RNA-seq-based, isoform-based, homology-based, and *de novo* predictions. The clean RNA-seq data were assembled into the *P. argenteus* genome using two different methods: (i) assembly with the reference genome using HISAT (v2.1.1)^61^, StringTie (v2.2.0)^62^, and Scallop2 (v1.1.1)^63^; and (ii) *de novo* assembly using RNA-Bloom (v2.0.1)^64^, Evigene^65^, minimap2 (v2.26)^66^ and miniprot (v0.12)^67^. For isoform-based prediction, SMRT-link (PacBio, USA) was used to generate isoforms and ESTs, and the transcriptome and protein sequences were generated with the Evigene^65^ platform; these sequences were subsequently mapped onto the *P. argenteus* genome using minimap2^66^ and miniprot^67^. Protein sequences from *D. dactyliophorus*^24^, *H. zosterae*^25^, *S. japonicus*^26^, *T. albacares*^27^, *T. maccoyii*^28^ and the Swissprot protein database^68^ were obtained from their genomes and aligned to the *P. argenteus* genome for homology-based gene prediction in miniprot^66^. The predicted gene models of RNA-seq, isoform, and homology-based strategies were used as training datasets in AUGUSTUS (v3.4.0)^69^ and SNAP (v2.0)^70^ for *de novo* prediction. Finally, all the predicted gene models were integrated into a single, non-redundant, and complete gene set using EvidenceModeler (v1.1.1)^71^. The untranslated region (UTR) and alternative splicing of these genes were annotated in Mikado (v2.3.2)^72^. The gene statistics, including gene length, coding sequence (CDS), intron length, and exon length, were similar between the reference^24–28^ and *P. argenteus* genomes (Fig. 5). The predicted protein-coding genes were annotated by searching the GenBank Non-Redundant (Nr) (ftp://ftp.ncbi.nih.gov/pub/nrdb/), SwissProt^68^, eukaryotic orthologous groups (KOG)^73^ and Kyoto Encyclopedia of Genes and Genomes (KEGG)^74^ protein databases using Diamond (v0.7.9)^75^, with an E-value threshold of 1e^−5^. EggNOG-mapper (v2.0)^76^ was used for gene ontology (GO) annotation in combination with the eggNOG database (v5.0)^77^. A total of 24,696 genes were predicted in the *P. argenteus* genome (Table 7). Among these, 23,062 genes (93.38%) were annotated by at least one database, while 12,974 genes (52.53%) were supported by all five databases (Table 8 and Fig. 6).

**Table 7:** Genes predicted in the *P. argenteus* genome using different methods.

Methods	Gene dataset	Number
*De novo*	Augustus	26326
	SNAP	32681
Homology-based	*H. zosterae*	26355
	*D. dactyliophorus*	29559
	*S. japonicus*	22978
	*T. albacares*	30887
	*T. maccoyii*	29936
	Swissprot	27540
Isoform-based	Miniprot (CDS)	34467
	Minimap2 (Exon)	42258
RNA-seq-based (Genome-guided assembly)	Miniprot (CDS)	46021
	Minimap2 (Exon)	73437
RNA-seq based (*De novo* assembly)	Scallop2 (CDS)	41129
	StringTie (Exon)	28574
Integration	EvidenceModeler	23148
Refine	Mikado	24696

**Table 8:** Gene function annotation statistics of the assembled genome for *P. argenteus*.

Database	Number of annotated gene	Percentage of predicted gene
GO	20,389	82.56%
KEGG	16,740	67.78%
KOG	15,916	64.45%
Swissprot	20,132	81.52%
Nr	22,857	92.55%
Total	23,062	93.38%

## Data Records

The Illumina (SRR27308594), PacBio HiFi (SRR27308592–SRR27308592), Hi-C (SRR27308591), RNA-seq (SRR27308587–SRR27308589) and isoform (SRR27308590) data used for the genome assembly of *P. argenteus* were deposited in the Sequence Read Archive (SRA) of the National Center for Biotechnology Information (NCBI) under sequence read project SRP479325^78^. The chromosome-level assembly of the *P. argenteus* genome was deposited in the NCBI genome database under accession number GCA_036321115^79^. The chromosome assembly of *P. argenteus*, genomic annotation results, and software settings can be found in the *figshare* database^80^.

## Technical Validation

### Evaluation of the genome assembly and annotation

The quality of this chromosome-level genome assembly was assessed using the following three criteria: (i) the mapping rate of Illumina PE150 reads, (ii) the Core Eukaryotic Genes Mapping Approach (CEGMA)^81^, and (iii) the Benchmarking Universal Single-Copy Orthologs (BUSCO) assessment^82^. In brief, 99.30% of the Illumina PE150 reads could be aligned to the *P. argenteus* genome using BWA (v0.7.12)^34^, for a coverage rate of 99.95%, which indicates high mapping efficiency and sufficient coverage. A total of 230 (92.74%) of the 248 highly conserved core genes for eukaryotes provided in CEGMA could be completely aligned with their homologous genes in the *P. argenteus* genome. In BUSCO (v4.1.2)^82^, 98.90% of the complete BUSCOs were detected in the *P. argenteus* genome, whereas fragmented and missing BUSCOs only comprised 1.08% of the total orthologs. This evidence indicated the high integrity and quality of the obtained chromosome-level genome assembly of *P. argenteus*.
